# Effect of Ghrelin on Caspase 3 and Bcl2 Gene Expression in H2O2 Treated Rat’s Bone Marrow Stromal Cells

**DOI:** 10.15171/apb.2018.050

**Published:** 2018-08-29

**Authors:** Alireza Abdanipour, Masoud Dadkhah, Mohsen Alipour, Hadi Feizi

**Affiliations:** ^1^Department of Anatomical Sciences, Faculty of Medicine, Zanjan University of Medical Sciences, Zanjan, Iran.; ^2^Department of Physiology and Pharmacology, Faculty of Medicine, Zanjan University of Medical Sciences, Zanjan, Iran.

**Keywords:** Ghrelin, Caspase 3, Bcl2, H2O2, Rat, BMSCs

## Abstract

***Purpose:*** The antiapoptotic effect of ghrelin in various cell lines including bone marrow stromal cells (BMSCs) has been proved. However, the real mechanism of this effect is not clear. Caspase3 and Bcl2 are well-known pro- and antiapoptotic regulatory genes in eukaryotes. The aim of the study was to find out the effect of ghrelin on Caspase 3 and Bcl2 change in BMSCs.

***Methods:*** Rat BMSCs were cultivated in DMEM. Passage 3 BMSCs were treated with ghrelin 100 μM for 48 h. Real-time PCR for Caspase 3 and Bcl2 was carried out from B (untreated BMSCs), BH (BMSCs treated with 125 µM H2O2), BGH (BMSCs treated with 100 µM ghrelin then 125 µM H2O2) and BG (BMSCs treated with 100 µM ghrelin) groups. For immunofluorescence, cells were incubated with anti Caspase 3 and Bcl2monoclonal antibodies. Primary antibodies were visualized using the FITC method. All data are presented as means ± SEM. Values of P<0.05 were considered statistically significant.

***Results:*** Ghrelin decreased mRNA expressions of Caspase-3 significantly as compared to the BH group (P<0.05). Also, Bcl-2 gene expression showed an increment in BG group as compare with BH and BGH groups (P<0.05). A high present of Bcl-2 positive cells were observed in the BGH group while Caspase-3 positive cells were significantly decreased in the BGH group compared with the BH group (P<0.05).

***Conclusion:*** Ghrelin probably enhances BMSCs viability through regulation of pro- and antiapoptotic genes Caspase 3 and Bcl2. However the signaling pathway of this effect should be elucidated in the future.

## Introduction

Ghrelin is an endogenous peptide that has some well known physiological functions especially in controlling the metabolism and food intake.^1^ It acts through a receptor belong to G protein-coupled receptors named GSR1α.^2^ This receptor has been found in different tissues including kidneys, adrenal glands, thyroid, breast, ovary, placenta, testis, prostate, liver, gallbladder, lung, skeletal muscles, myocardium, skin, and bone.^3^ Since its discovery, ghrelin has been shown to be involved in many physiological and pathophysiological roles such as regulation of glucose and lipid metabolism, modulation of immunity, stimulation of gastric motility, cardiovascular function, modulation of appetite, stress, anxiety, taste sensation and behavior in nervous system, as well as metabolic complications, chronic inﬂammation, gastroparesis or cancer-associated anorexia and cachexia.^4,5^ One of the recently introduced roles of ghrelin is the antiapoptotic and cell injury protection.^6^

Bone marrow stromal cells (BMSCs) are a population of progenitor cells for skeletal tissue constituents.^7^ These cells are capable to differentiate into bone, cartilage, and adipocytes.^8^ BMSCs also support hematopoietic stem cells structurally and physiologically.^9^ BMSCs have been applied in several cell therapy strategies in order to tissue repair and functional recovery.^10,11^ Therefore, autologous BMSCs can be isolated from bone marrow and used as a credible source of stem cells for restoring injured tissue function. However, previous studies have shown that transplanted BMSCs do not accommodate well within diseased tissues.^12^ There is evidence that these cells are suffered due to host immune responses and die because of apoptosis.^13^

Apoptosis is a programmed cell-suicide in which some gene products are responsible as apoptotic effectors proteins and some of them act as antiapoptosis regulators.^14^ Caspases are a gene family that acts in a cascade manner and caspase3 is one of the final effectors leading to apoptosis.^15^ Among the anti‏-apoptotic genes, Bcl2 is considered to be one of the most important and well-known genes.^16^ Due to the apoptosis inducers, oxidative stress is a common cause and H2O2 is a mediator of this phenomenon.^17^

Recently we have shown that ghrelin increases the BMSCs viability and protect them against the H2O2 induced damage.^18^ Consequently, using ghrelin, as an endogenous peptide, that enhances BMSC’s resistance to apoptosis would improve the therapeutic potential of these cells. However, to find out the mechanism of this phenomenon, in the present study we are going to examine the probable effects of this peptide on the Caspase 3 and Bcl2 gene expression in H2O2 treated rat’s BMSCs.

## Materials and Methods

### BMSCs culture and drug treatments

Male Wistar rat of 4-6 weeks were sacrificed under deep anesthesia using ketamine–xylazine (K, 100 mg/kg; X, 10 mg/kg). The lower limbs were removed with a pair of scissors separating it from the hip joint and put on a sterile gauze. The accompanied soft tissue (muscles, fasciae, and tendons) was removed, and femurs and tibiae were separated and put in a dish containing phosphate buffered saline (PBS, Gibco, Life Technologies, USA) and penicillin/streptomycin (Gibco, Life Technologies, USA). The dish was transferred under a laminar hood. The bones were subsequently washed again with PBS and put on a sterile gauze to dry. Both ends of the bones were cut, then with an insulin syringe containing high glucose Dulbecco’s Modified Eagle Medium (DMEM, Gibco, Life Technologies, USA) and 1% penicillin/streptomycin, all the contents of the bone’s lumen were flushed directly to 25 cm2 culture flask (SPL, life sciences, Korea) without any additional manipulation. The flushing was done several times, so that the lumen became pale. Rat BMSCs were initially cultivated in DMEM (Dulbecco's Modified Eagle Medium), supplemented with 20% FBS (Gibco), 100 U/ml penicillin, and 100 mg/ml streptomycin in 4 experimental groups as B (untreated BMSCs), BH (BMSCs treated with 125 µM H2O2), BG (BMSCs treated with 100 µM ghrelin) and BGH (BMSCs treated with 100 µM ghrelin then 125 µM H2O2). The cells were incubated at 37°C (5% CO2) in 25 cm2 plastic flask. The medium refreshed every 2-3 days until cells became confluent. The cells were harvested with trypsin–EDTA and passaged up to three times. To induce BMSC, ghrelin was freshly prepared. Passage 3 BMSCs were cultured in 96-well plates (5000 cells/well) in DMEM medium supplemented with different concentration of ghrelin (0.1, 1, 10 and 100 μM) for 24 and 48 h.

### Real-time PCR

Real-time PCR was carried out with RNA from B (untreated BMSCs), BH (BMSCs treated with 125 µM H2O2), BGH (BMSCs treated with 100 µM ghrelin then 125 µM H2O2) and BG (BMSCs treated with 100 µM ghrelin) groups. In all groups, 1,000 ng purified RNA from cultured cells was used to synthesize 20 μlcDNA, using Revert aid™ first strand cDNA synthesis kit (Fermentas, Germany) according to the manufacturer’s instructions. cDNA (25ng) was used to quantify Caspase3 and Bcl2 mRNA levels. As an internal control, primers for GAPDH were used. All primers have been listed in Table 1. The PCR reaction was synthesized in a 12.5μl volume (sense and anti-sense primers, cDNA, Sybr green,) and carried out for 40 cycles (Applied Biosystems cycler). For analyzing relative changes in mRNA levels, we used the delta CT method (Pfaffl method).

**Table 1:** Sequences of Oligonucleotide Primers

**Name**	**Sequence (5' → 3')**
***Caspase3*** **(Forward)**	GGTATTGAGACAGACAGTGG
***Caspase3*** **(Reverse)**	CATGGGATCTGTTTCTTTGC
**Bcl2 (Reverse)**	ATCGCTCTGTGGATGACTGAGTAC
**Bcl2 (Reverse)**	AGAGACAGCCAGGAGAAATCAAAC
**GAPDH (Forward)**	CAAGGTCATCCATGACAACTTTG
**GAPDH (Reverse)**	GTCCACCACCCTGTTGCTGTAG

### Immunostaining

BMSCs were cultured on cover slides and fixed in 3% paraformaldehyde for 20 min at RT, followed by a permeabilization step in 100% methanol for 30 min at RT (room temperature). For immunofluorescence, cells were incubated with anti-CD90 (for BMSCs) and Anti-Caspase3 and Bcl2 (for produced erythroid Progenitor Cells) monoclonal antibodies, followed by incubation with a fluorescein isothiocyanate (FITC)–conjugated Rabbit anti-Mouse antibody (millipore). Nuclei were counterstained with DAPI. For indirect immunoperoxidase labeling, 100 µM treated BMSCs (for 48 h) were permeabilized with 0.4% Triton X-100, followed by FCS 10% for 60 minutes to block endogenous peroxidase. Then were incubated with anti-CD90 and Caspase3 and Bcl2 antibodies overnight at 4°C. Primary antibodies were visualized using the FITC method.

### Statistics

Statistical analysis was performed using the SPSS15 software. All data are presented as means ± SEM. To compare multiple means in groups, one-way ANOVA followed by Tukey's post hoc comparison was used. Values of P<0.05 were considered statistically significant.

## Results

### BMSCs expansion and identification

**Figure 1:**
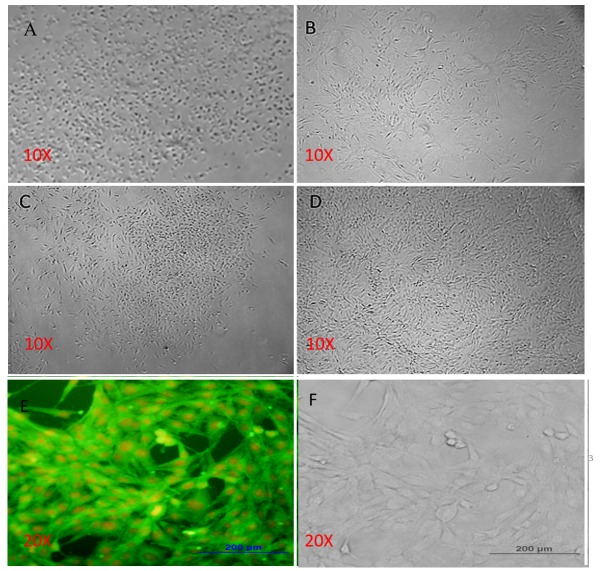
Micrographs of bone marrow stromal cells (BMSCs).**A**in primary culture the BMSCs had round shapes (after 12 hrs). **B;** The cells are fibroblast-like cells after 48 hours. **C;** Cells at the stage of the first passage and formation of colonies. **D;** BMSCs have a more uniform spindle shape after 4 passages. **E, D;** represents Phase contrast micrographs of BMSCs and immunostaining of CD90 at same field respectively. The cells were immunostained with relevant primary antibodies and labeled with FITC-conjugated secondary antibody (green color shows positive cells) and the red colors are ethidium bromide counterstaining of the nuclei

The Ethics Committee for animal studies at the University of Zanjan University (ZUMS) confirmed the experiment conducted in this study. The primary culture of the isolated BMSCs is presented in Figure 1-A-D. The results showed, after 12 hours, the cells were attached to the flask and most of them were rounded (Figure 1-A). Adherent cells were cultured and became heterogeneous after 12 or 16 days (passage 4) (Figure 1-D). Following, the cells were immunostained with anti-CD90 (mesenchymal stem cells markers) antibody and incubated with FITC conjugated secondary antibody. The result showed, 100% of the cells were immunoreactive to CD90 (Figure 1-E, F).

### Bcl-2 and Caspase-3 genes expression rates

**Figure 2:**
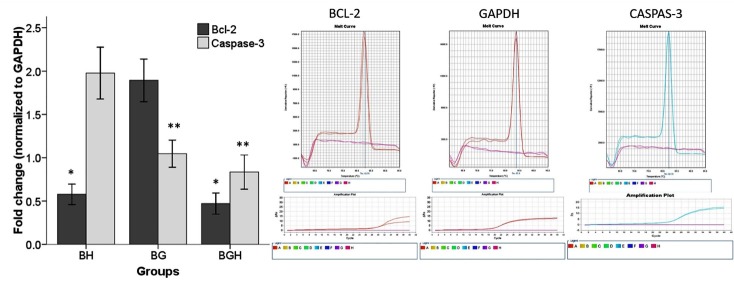
Bcl-2 and Caspase-3 genes expression. Fold change ratio of *Bcl-2* and *Caspase-3*mRNA of BMSCs treated with 100 µM concentrations of Ghrelin for 48 hrs and various experimental groups. Real-time PCR results have been presented as relative expression normalized to GAPDH mRNA amplification. Amplification of the Bcl-2 and Caspase-3 mRNA derived from BH, BG and BGH groups showing increases level of Bcl-2 mRNA and decreasing *Caspase-3*mRNAin the BG and (BG, BGH) groups respectively. The bars indicate the mean ± SEM. P<0.05 *(compared to BG group), **(compared to BH group). B (untreated BMSCs), BH (BMSCs treated with 125 µM H2O2), BG (BMSCs treated with 100 µM ghrelin) and BGH (BMSCs treated with 100 µM ghrelin then 125 µM H2O2)

Decreasing of both genes expressions in BGH and other groups (BH and BG) at 48 hrs were confirmed by quantitative real-time RT-PCR. The results of the mRNA expression pattern have been shown in the (Figure 2). Our data showed that mRNA expressions of ***Caspase-3 ***gene significantly decreasing when Ghrelin was used (BGH;0.83 ± 0.09, BG; 1.04 ± 0.07) as compare to the BH group (1.97 ± 0.14). Also, the result showed, increasing of the Bcl-2 gene in BG group (1.89 ± 0.12) as compare with BH (0.57 ± 0.05) and BGH (0.47 ± 0.06).

### Immunostaining of Bcl-2 and Caspase-3 

**Figure 3:**
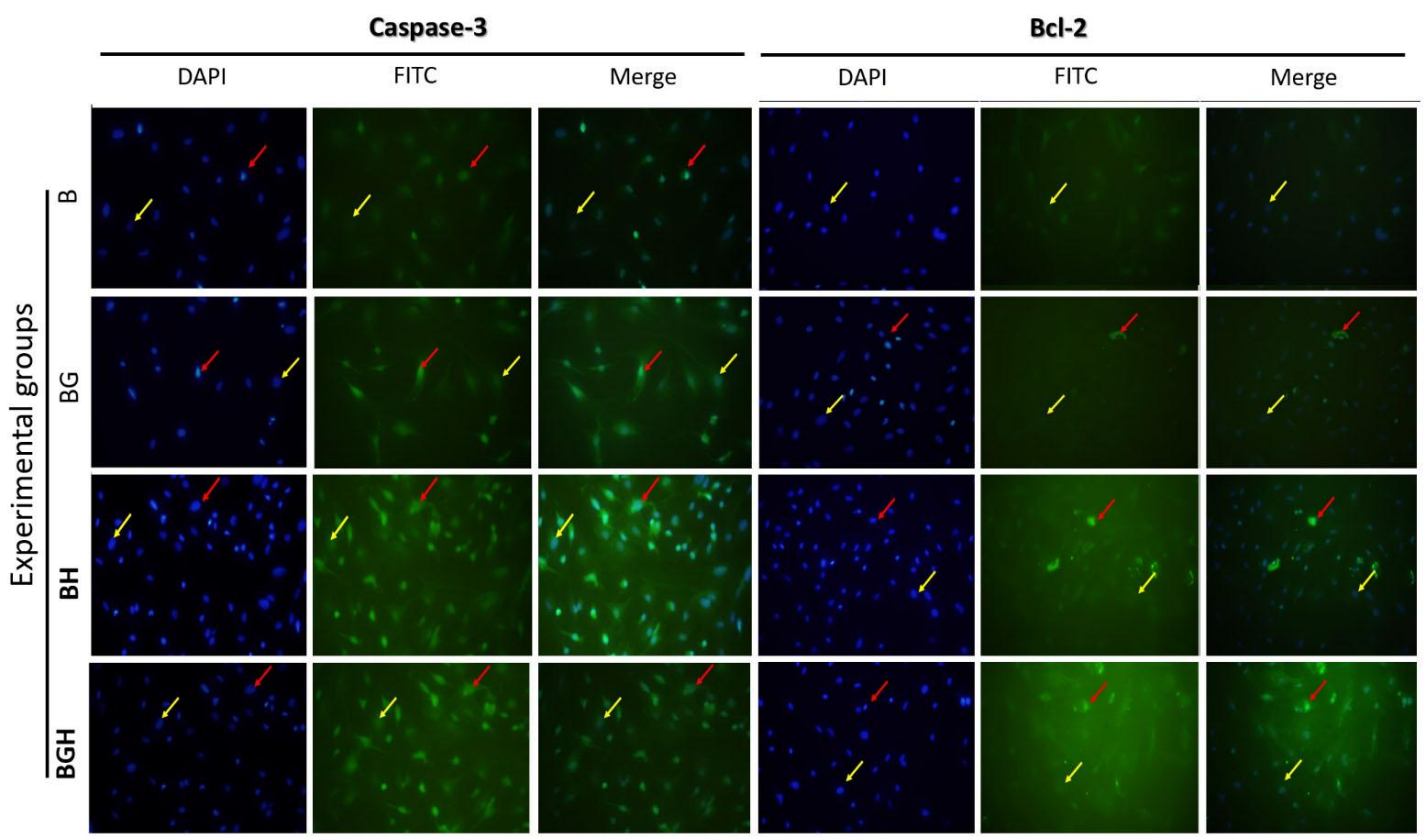
*Bcl-2* and *Caspase-3* protein expression. Representative immunostaning-photomicrographs showing Bcl-2 (left panel) and Caspase-3 (right panel) immunoreactivity in the B, BG, BH and BGH experimental groups after 48 hrs of treatments. Red arrows indicate to immunopositive cells and yellow arrows indicate to negative cells. Magnification, 200×. B (untreated BMSCs), BH (BMSCs treated with 125 µM H2O2), BG (BMSCs treated with 100 µM ghrelin) and BGH (BMSCs treated with 100 µM ghrelin then 125 µM H2O2)

**Figure 4:**
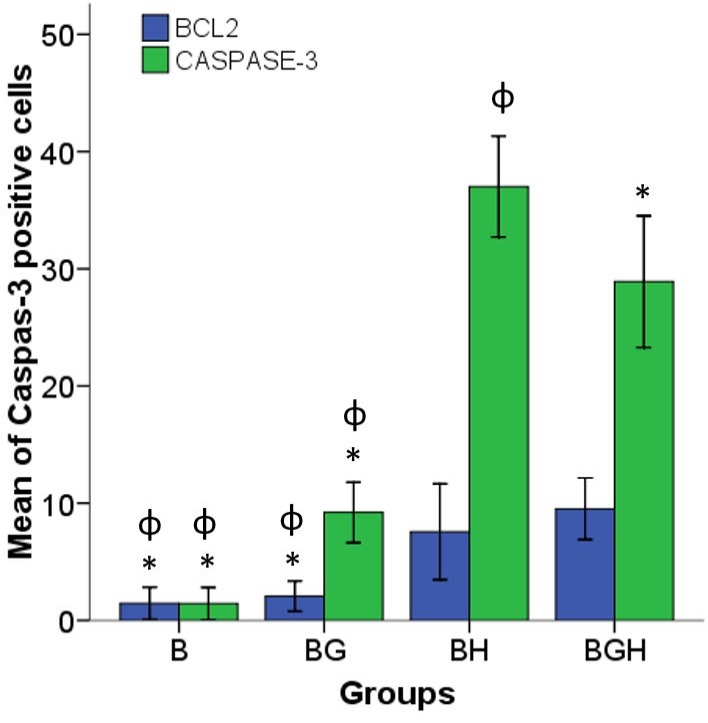
represents the histogram of the mean percentage of the Bcl-2 and Caspase-3 protein positive cells in the B, BG, BH and BGH experimental groups. The bars indicate the mean ± SEM; P<0.05,*(compared to BH group), Ω (compared to BGH group). B (untreated BMSCs), BH (BMSCs treated with 125 µM H2O2), BG (BMSCs treated with 100 µM ghrelin) and BGH (BMSCs treated with 100 µM ghrelin then 125 µM H2O2)

To determine the protective effect of Ghrelin, *Bcl-2* and *Caspase-3* protein expression were detected using immunocytochemistry technique. The results were shown in the Figures 3 and 4. The percentage of *Bcl-2* (Figure 3 left panel) and *Caspase-3* (Figure 3 right panel) Positive cells were calculated in 5 samples. A high present of *Bcl-2* (9.52 ± 1.31) and *Caspase-3* (37.01 ± 2.15) positive cells were observed in the BGH and BH groups respectively. But the low percentage of *Bcl-2* (1.46 ± 0.68) positive cells were visible in the B group. The percentage of *Caspase-3* positive cells was significantly decreased in the BGH group (26.09 ± 2.8) compared with the BH group (37.02 ± 2.15).

## Discussion

According to the results of the current study, ghrelin (100µM) significantly decreased both gene expression and protein production of Caspase-3 in H2O2 suffered BMSCs. Ghrelin treatment also enhanced BCl2 production in these cells. As mentioned previously we have detected an antiapoptotic effect for ghrelin in BMSCs during similar condition.^18^ Thus the presented results could justify our former finding. It has been proved that H2O2 induces apoptosis in BMSCs and this injury could be restored by melatonin via Bax/Bcl-2 ratio suppression and caspase-3 inactivation.^19^ Our results are consistent with the following studies in the text however they have been performed in different cells and treatment situations.

It has been demonstrated by Baldanzi et al. that ghrelin inhibits cell death in cardiomyocytes and endothelial cells and they showed that this effect was through ERK1/2 and PI 3-kinase/AKT pathways.^20^ As reported by Yang et al., ghrelin repressed apoptosis signal-regulating kinase 1 activity in PC12 cells and thus caspase 3 inhibitions through heat-shock protein 70 upregulation.^21^ It has been shown that ghrelin inhibits apoptosis in pancreatic β cell line HIT-T15. This effect was achieved via activation of MAPK and Akt pathways. Ghrelin also increased Bcl-2, decreased Bax, and suppressed caspase-3 activation in this cell.^22^ Moreover, it has been revealed that ghrelin (1000 ng/ml) in a dose-dependent manner inhibits TNF-alpha-induced apoptosis of vascular smooth muscle cells.^23^ Previous studies have shown that ghrelin treatment diminishes diabetes-induced cell death in lactotrophs through caspase-8 inhibition and increasing Bcl-2 levels.^24^

Bando and coworkers indicated that streptozotocin treated transgenic (RIP-GG Tg) mice, which have elevated pancreatic ghrelin levels, showed a significant elevation in pancreatic insulin mRNA expression. Furthermore, β-cell numbers increased in islets.^25^ Han and colleagues have shown that ghrelin administration (10−8^−8^M) combined with intramyocardial injection of adipose-derived mesenchymal stem cells (ADMSCs) inhibited cardiomyocyte apoptosis. Ghrelin increased ADMSCs survival under hypoxia/serum deprivation (H/SD) injury. It also decreased the proapoptotic protein Bax and increased the antiapoptotic protein Bcl-2 in vitro, and these effects were eliminated by PI3K inhibitor LY294002.^26^ Furthermore, it has been reported that ghrelin could reverse rotenone-induced neurotoxicity in MES23.5 cells through improving the mitochondrial dysfunction and finally inhibition of caspase-3 activation and apoptosis.^27^ In a study by Zhang and his group, ghrelin (0.1 μM) inhibited dexamethasone-induced apoptosis in INS-1 cells. It upregulated Bcl-2 and downregulated Bax expression, and decreased caspase-3 activity. Moreover, this protective effect of ghrelin was through GHS-R1a and the ERK and p38MAPKsignaling pathways.^28^ HOXb4 is one of the factors that its upregulation, especially in hematopoietic cells, protects them against apoptosis.^29^ Recently we have shown that ghrelin upregulates HOXB4 gene expression in the rat BMSCs.^30^ These mentioned in vitro studies which imply the antiapoptotic effect of ghrelin are matching with some in vivo studies.^31-33^ It has been identified that ghrelin causes an antiapoptotic effect in the renal tissue of chronic hypoxic rats by increasing the Bcl2/Bax ratio.^34^

A couple of studies have shown the therapeutic potential of BMSCs.^35^ For example, BMSCs administration recovers neural tissue injury.^36,37^ Their involvement in bone regeneration also has been identified.^38^ Further investigations have shown the BMSCs beneficiary in renal injuries.^39,40^ Ghrelin has been shown to be protective against multiple complications in various cells.^21-28^ However, its effect on bone marrow stem cells has not been investigated prior to this study. We demonstrated that BMSCs treated with ghrelin are less vulnerable to oxidative stress.^18^ The physiological function of endogenous ghrelin in BMSCs is not clear. In the present report, the authors suggest that ghrelin changes the expression of Bcl-2 and Caspase3 under H2O2-induced stress and this may regulate BMSCs survival. Since ghrelin is an endogenous peptide with the fewer side effects, its application as co-treatment in the medium could be valuable in developing the cell therapy strategies.

## Conclusion

Ghrelin probably enhances BMSCs viability through regulation of pro- and antiapoptotic genes Caspase 3 and Bcl2. However, the signaling pathway and in vivo application of this effect should be elucidated in future.

## Acknowledgments

The results described in this paper were part of student thesis (Masoud Dadkhah) for MSc degree in physiology. The authors would like to thank the Vice-Chancellery for Research affairs of Zanjan University of Medical Sciences for financial support (grant no.A-10-141-7).

## Ethical Issues

All the experiments were carried out under the ethical guidelines of Zanjan University of Medical Sciences (ZUMS.REC.1394.147).

## Conflict of Interest

The authors report no conflicts of interest. The authors alone are responsible for the content and writing of the paper.
